# Longitudinal patterns of mental well-being over four years in a german general population sample: a growth mixture modeling approach

**DOI:** 10.1186/s12889-025-23539-w

**Published:** 2025-06-26

**Authors:** Johanna Fischer, Ulrich John, Hans-Jürgen Rumpf, Andreas Staudt, Sophie Baumann

**Affiliations:** 1 Institute and Policlinic of Occupational and Social Medicine, Faculty of Medicine, Technische Universität Dresden, Fetscherstr. 74, 01307 Dresden, Germany; 2 Department of Prevention Research and Social Medicine, Institute for Community Medicine, University Medicine Greifswald, Walther-Rathenau-Str. 48, 17475 Greifswald, Germany; 3 Department of Psychiatry and Psychotherapy, Research Group S:TEP, University of Lübeck, Ratzeburger Allee 160, 23538 Lübeck, Germany; 4 Department of Methods in Community Medicine, Institute for Community Medicine, University Medicine Greifswald, Walther‑Rathenau‑Str. 48, 17475 Greifswald, Germany

**Keywords:** Mental health, Mixture modeling, Longitudinal patterns of mental well-being, MHI-5, Latent trajectory class, Lifestyle behaviors

## Abstract

**Background:**

Although mental well-being is facing growing challenges against the background of global crises such as climate change, pandemics, and social inequality, little is known about longitudinal patterns of mental well-being in the general population.

**Methods:**

The study is based on self-report data from 1,605 adults aged 18 to 64 years who were proactively recruited at a municipal registration office (*M* = 31.0 years, *SD* = 10.8 years). Mental well-being was assessed at baseline, and 3, 6, 12, 36 and 48 months later using the five-item Mental Health Inventory (MHI-5). Covariates were smoking, alcohol consumption, fruit and vegetable intake, moderate-to-vigorous physical activity, age, sex, school education and relationship status. Growth Mixture Modeling was used to identify latent trajectory classes of mental well-being. Multinomial logistic regression was used to test whether class membership is predicted by health behaviors and sociodemographic variables.

**Results:**

Three latent trajectory classes were found. The first class (“stable high”, *n* = 1,251, 78%) showed the highest mental well-being throughout the study with only minor fluctuations over time. The second class (*n* = 192, 12%) showed a “steadily increasing” trajectory starting with the lowest MHI-5 sum score of the three classes at baseline that increased over time. The third class (“fluctuating”, *n* = 162, 10%) reported a slight increase in mental well-being during the first six months, followed by a steep decline to 36 months, which then increased to almost baseline-level at 48 months. The odds of being classified into the “steadily increasing” or “fluctuating” compared to the “stable high” class were higher for men and younger participants. The odds of belonging to the “steadily increasing” compared to the “stable high” class were higher for participants with more than 12 years of school education and those being in a relationship. Health behaviors did not predict latent trajectory class membership.

**Conclusion:**

Three different temporal patterns of mental well-being were found in a German general population sample, with the majority showing a stable level of high mental well-being over four years. Further research is needed to understand fluctuating patterns and the causal factors influencing mental well-being.

**Supplementary Information:**

The online version contains supplementary material available at10.1186/s12889-025-23539-w.

## Background

Mental well-being is a central component of general health [1] and encompasses a dynamic equilibrium between physical and psychological health, which is reinforced by the development of social and emotional competencies [2]. The World Health Organization defines mental health as a state of well-being that enables individuals to recognize their abilities to cope with everyday challenges, work productively, and contribute to the community [1, 3]. It is a fundamental human right and essential for an individual’s quality of life, social participation and performance [4, 5]. Mental well-being can be defined as a person’s subjective assessment of their current mental condition, closely associated with emotions and life satisfaction. While mental well-being is a component of mental health, it is possible to experience poor mental well-being, without fulfilling diagnostic criteria for psychiatric disorders [6].

It is estimated that approximately 970 million individuals worldwide are affected by mental illness, constituting a substantial challenge for public health [7] and a major contribution to the global burden of disease [8, 9]. A report by a large statutory health insurance company has indicated that the number of sick days attributable to mental stress in Germany has increased by 50% over the past decade [10].

There are only few studies that have investigated long-term patterns of mental well-being and the effects of various influencing factors on these patterns [11, 12]. Despite the establishment of initiatives such as the German mental health surveillance system, the available evidence is mainly limited to cross-sectional analyses [13]. To establish effective targeted prevention, it is necessary to adopt a multidimensional approach that integrates sociodemographic factors and individual behaviors [11, 12]. Furthermore, longitudinal studies are required to analyze the long-term effects of these influencing factors on mental well-being [14]. The findings would provide a valuable basis for evidence-based interventions that are aiming to promote and preserve mental well-being in the population [13].

Health behaviors such as smoking, alcohol consumption, fruit and vegetable intake, and physical activity can have an impact on individuals’ mental well-being [15, 16]. There may be a reciprocal relationship between lifestyle and mental well-being. On the one hand, an unhealthy lifestyle has been associated with a decline in mental well-being [17, 18], as evidenced by findings related to smoking [19–21], alcohol consumption [22, 23], unhealthy eating habits [21, 24, 25], and low physical activity [26]. In contrast, nicotine or alcohol may be consumed to cope with stress or anxiety. The self-medication hypothesis posits that poor mental well-being may lead to increased smoking or alcohol use [21, 24, 25]. A healthy lifestyle characterized by regular consumption of fruits and vegetables [27, 28] and moderate-to-vigorous physical activity [15, 29] has been found to improve quality of life and mental well-being.

Sociodemographic factors such as age, sex, school education and being in a relationship can affect mental well-being, too [30–34]. A higher prevalence of mental disorders has been observed among women [35], who also reported higher levels of stress compared to men [36]. Older people are affected by stressful life events more frequently, which can lead to impaired mental well-being [37]. Although 18-to-29-year-olds have been shown to be optimistic, energetic and positive, they may also encounter elevated levels of psychological distress [32, 37]. Individuals with higher levels of education have been shown to have a lower risk of illness and higher life expectancy, likely as a result of healthier lifestyles [38] or better access to resources, and resilience [8, 39], potentially affecting their mental well-being.

In order to fill the research gap regarding the longitudinal patterns of mental well-being, the aim of this study was to identify latent trajectory classes of mental well-being over four years in a German general population sample. Furthermore, we aimed to investigate to what extent these trajectory classes are predicted by health behaviors and sociodemographic variables.

## Methods

This analysis is based on the “Testing a proactive expert system intervention to prevent and to quit at-risk alcohol use (PRINT)” study, a randomized-controlled trial evaluating a brief alcohol intervention consisting of three theory-based and individualized feedback letters [40]. The study was prospectively registered in the German Clinical Trials Register (DRKS00014274, registration date: 12th March 2018). Primary and secondary outcome data and findings on reach and retention have been published elsewhere [41–43].

### Study population and procedures

The study participants were recruited between April and June 2018 in the waiting area of the registry office in Greifswald, Mecklenburg-Western Pomerania, Germany. All visitors to the municipal registry office were proactively approached by members of the study team and invited to take part in a short electronic self-administered health survey. People were excluded if they were younger than 18 or older than 64 years, if they were physically or cognitively impaired, if they had insufficient German language skills, if they had been approached on a prior visit to the registration office, or if they were employed at the institute conducting the study. Of 3,969 eligible visitors, 2,947 completed the initial health survey (participation rate: 74%), which included the screening procedure for the PRINT trial. Those who reported alcohol consumption in the past 12 months received extensive information from the study assistants and were asked to participate in the trial. Individuals without permanent address or telephone number had to be excluded. Of 2,462 eligible individuals, 1,646 provided written informed consent (participation rate: 67%) and were randomized to either intervention or control group in a 1:1 allocation ratio. All participants were assessed at 3 (July to September 2018), 6 (October 2018 to January 2019), 12 (April to June 2019), 36 (April to June 2021), and 48 months (April to June 2022) via standardized, computer-assisted telephone interviews. Those who were not reached after ten unsuccessful attempts received questionnaires by e-mail of post. Participation in the follow-ups was 85% (3 months), 81% (6 months), 80% (12 months), 65% (36 months), and 59% (48 months), respectively.

The intervention group received up to three computer-generated individualized feedback letters at baseline, after three and six months. These letters addressed the participants’ alcohol consumption and motivational variables according to the Transtheoretical Model of Behavior Change [44]. The control group received assessment-only. More detailed information on the intervention and comparator can be found elsewhere [43].

### Measures

#### Mental well-being

Mental well-being was assessed using the five-item Mental Health Index [MHI-5]; [45]. The MHI-5 contains five questions: “How much of the time during the last month have you…”, “…been a very nervous person”, “…felt calm and peaceful”, “…having felt downhearted and blue”, “…been a happy person”, and “…felt so down in the dumps that nothing could cheer you up”. The responses were recorded on a five-point Likert scale (1 = “never”, 2 = “rarely”, 3 = “occasionally”, 4 = “often”, 5 = “always”). A five point scale was originally implemented for the MHI-5 to harmonize the answer format of the assessment tools which proved validity in a general population study [46]. The raw sum scores (ranging from 5 to 25) were standardized by linear transformation to a scale between 0 and 100, with higher scores indicating better mental well-being [45]. The MHI-5 sum score was used in the present study as a continuous measure of mental well-being, showing acceptable internal consistency with Cronbach’s alpha ranging between 0.76 and 0.81. To our knowledge, there is no single universally accepted gold standard for measuring mental well-being in population studies. The MHI-5 was derived from the longer Mental Health Inventory and is particularly suitable for longitudinal population studies, balancing psychometric quality and feasibility due to the limited number of items [45, 47]. Originally, the MHI-5 was developed as a screening tool for mood and anxiety disorders. According to the Receiver Operating Characteristic (ROC) curves, MHI-5 values below 60 were suggested for identifying mood disorders, and values below 70 for anxiety disorders, facilitating the differentiation between varying levels of psychological distress [45].

#### Covariates

All health behaviors were assessed at baseline. Participants were asked if they currently smoked. Responses were condensed into a dichotomous variable differentiating nonsmokers (lifetime nonsmokers and former smokers) and smokers (occasional and daily smokers). Alcohol consumption was assessed using the three-item Alcohol Use Disorders Identification Test-Consumption [AUDIT-C; 48]. Respondents were asked how often they have a drink containing alcohol, how many alcoholic drinks they consume on a typical drinking day, and how often they have 4 or more (women) or 5 or more (men) alcoholic drinks on a single occasion. The AUDIT-C sum score was used with higher values reflecting higher levels of alcohol consumption. Healthy eating was operationalized as the number of portions of fruits and vegetables eaten on a typical day. Participants received the information that one portion is understood as “an apple, a small bowl of salad or a handful of vegetables as a side dish” and entered their answer in a free number field. Moderate-to-vigorous physical activity (MVPA) was assessed separately for weekdays and weekend days. Participants were asked to indicate the time they spent in MVPA on a typical day during the week (Monday through Friday) and during the weekend (Saturday and Sunday). The definition of MVPA as any activity that “involves a marked acceleration in heart rate and breathing, such as brisk walking, jogging, cycling, swimming, or gardening” was based on prior operationalizations [49, 50]. Only MVPA during leisure time (including travel to and from work or from one place to another) were referred to as permissible information. For the data analysis, weekdays and weekend days were combined into one variable containing the average time spent in MVPA per day in minutes.

Sex was assessed as binary variable (0 = male, 1 = female). Age was assessed as continuous variable. Relationship status (0 = currently not in a relationship, 1 = currently in a relationship) was derived from two questions asking participants to disclose their marital and relationship status. Educational background was derived from participants’ self-reported highest general educational degree. During the assessment, an exhaustive list of possible German and foreign school-leaving qualifications was presented, out of which participants could choose. The responses were condensed into a binary variable of school education (0 = less than 12 years, 1 = 12 or more years of school education).

### Statistical analysis

**Fig. 1:**
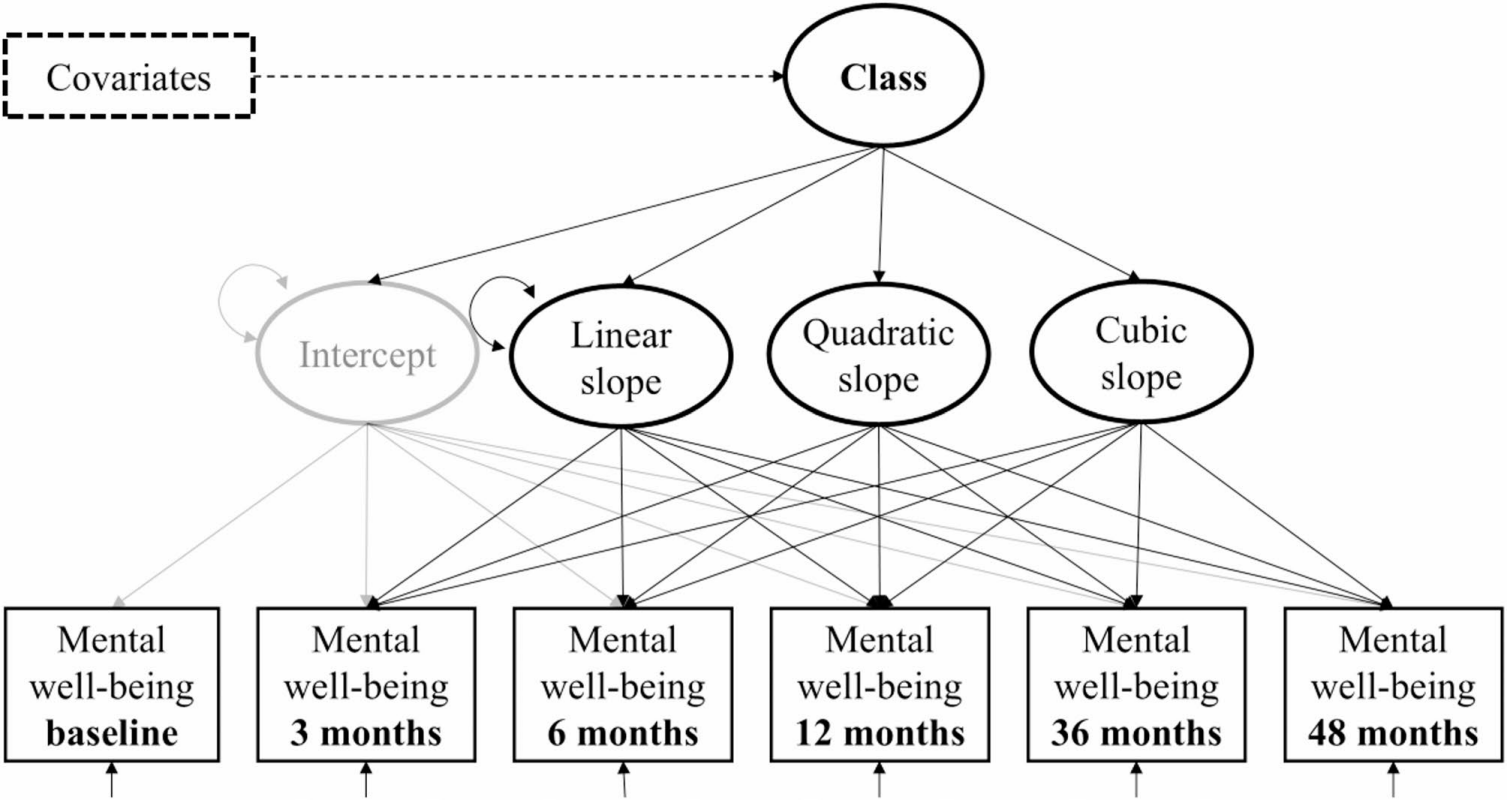
Growth Mixture Model for latent trajectory classes of mental well-being. *Note*. Growth Mixture Model for latent trajectory classes of mental well-being. Mental well-being as repeatedly observed manifest variable over time (in rectangles) is accounted for by linear, quadratic, and cubic growth factors. Heterogeneity in the mental well-being trajectories is captured by a latent class variable that is predicted by sociodemographic variables and health behaviors. The three-step approach for incorporating covariates into mixture models was applied [54]

Growth Mixture Modeling (GMM) was used in Mplus version 8.8 [51]. The descriptive analyses were done in RStudio version 9.1 [52]. The aim was to identify distinct longitudinal patterns of mental well-being. In a GMM, changes in a repeatedly measured variable are captured by latent growth factors (Fig. 1) [53]. A categorical latent class variable is used to incorporate unobserved heterogeneity in the development over time. The probability of being assigned to a specific latent class can be predicted in a multinomial logistic regression model. Missing data was handled by full-information maximum likelihood estimation. A total of 41 participants with missing data on all MHI-5 sum scores had to be excluded from the analysis, resulting in a final sample size of 1,605 participants.

The data were analyzed in successive steps. First, different models were compared to find the structural model and constellation of latent growth factors that best described the development of mental well-being over time. The model fit statistics included the Comparative Fit Index (CFI), Tucker-Lewis Index (TLI), Root Mean Square Error of Approximation (RMSEA), and Standardized Root Mean Square Residual (SRMR). The models were compared using a chi-square test to determine which model had a significantly better fit. These preliminary analyses revealed that a cubic model with the variances of the cubic and quadratic growth factor fixed at zero (*CFI* = 0.96, *TLI* = 0.96, *RMSEA* = 0.06, *SRMR* = 0.05) provided the best fit (see also Fig. 1). Second, models with two to five latent classes were compared using entropy, Akaike Information Criterion (AIC), Bayesian Information Criterion (BIC), class sizes and average latent class posterior probabilities (LCPP), as well as *p*-values of the Vuong-Lo-Mendell-Rubin likelihood ratio test (VLMR). Third, the latent class variable was regressed on sociodemographic variables (age, sex, education, relationship) and health-related behaviors (smoking, AUDIT-C, fruit and vegetable intake, MVPA) using multinomial logistic regression. The regression was adjusted for study group membership (intervention or assessment-only control group) to control for potential bias due to differences between groups. The three-step approach for incorporating covariates into mixture models was applied [54], meaning that the latent trajectory classes were only formed by the repeatedly measured MHI-5 sum score over time. The classification uncertainty was taken into account when predicting latent class membership through the latent class posterior distribution. This ensured that the latent trajectories of mental well-being and the participants’ class assignment remained unchanged after the addition of covariates compared to the initial model without covariates. Results of the logistic regression are given as Odds Ratios (ORs) and 95% Confidence Intervals (95% CI).

## Results

### Sample

The sample encompassed 1,605 participants (Table 1), more than half of them were female (*n* = 893, 56%), the mean age was 31.0 years (SD = 10.8). The majority (*n* = 1,509, 94%) had at least ten years of school education, more than half (*n* = 1,052, 66%) were in a relationship, and there were fewer smokers (*n* = 509, 32%) than non-smokers (*n* = 1,096, 68%). The average AUDIT-C score was 3.51 (*SD* = 1.8). Participants ate an average 2.2 portions of fruit and vegetables per day (*SD* = 1.4). The average daily time spent in MVPA was 73.6 min (*SD* = 81.2), and the baseline MHI-5 sum score was 66.8 (*SD* = 15.9).

### Longitudinal patterns of mental well-being

AIC and BIC decreased with increasing number of classes (Table 2). The estimation of models specifying more than three classes led to very small class sizes and the best loglikelihood values could not be replicated, even with a high number of random starting values. The VLMR *p*-value favored the three-class model. Therefore, the three-class model was selected for further analysis. Entropy was 0.78 and the lowest average latent class posterior probability was 0.83.

**Fig. 2:**
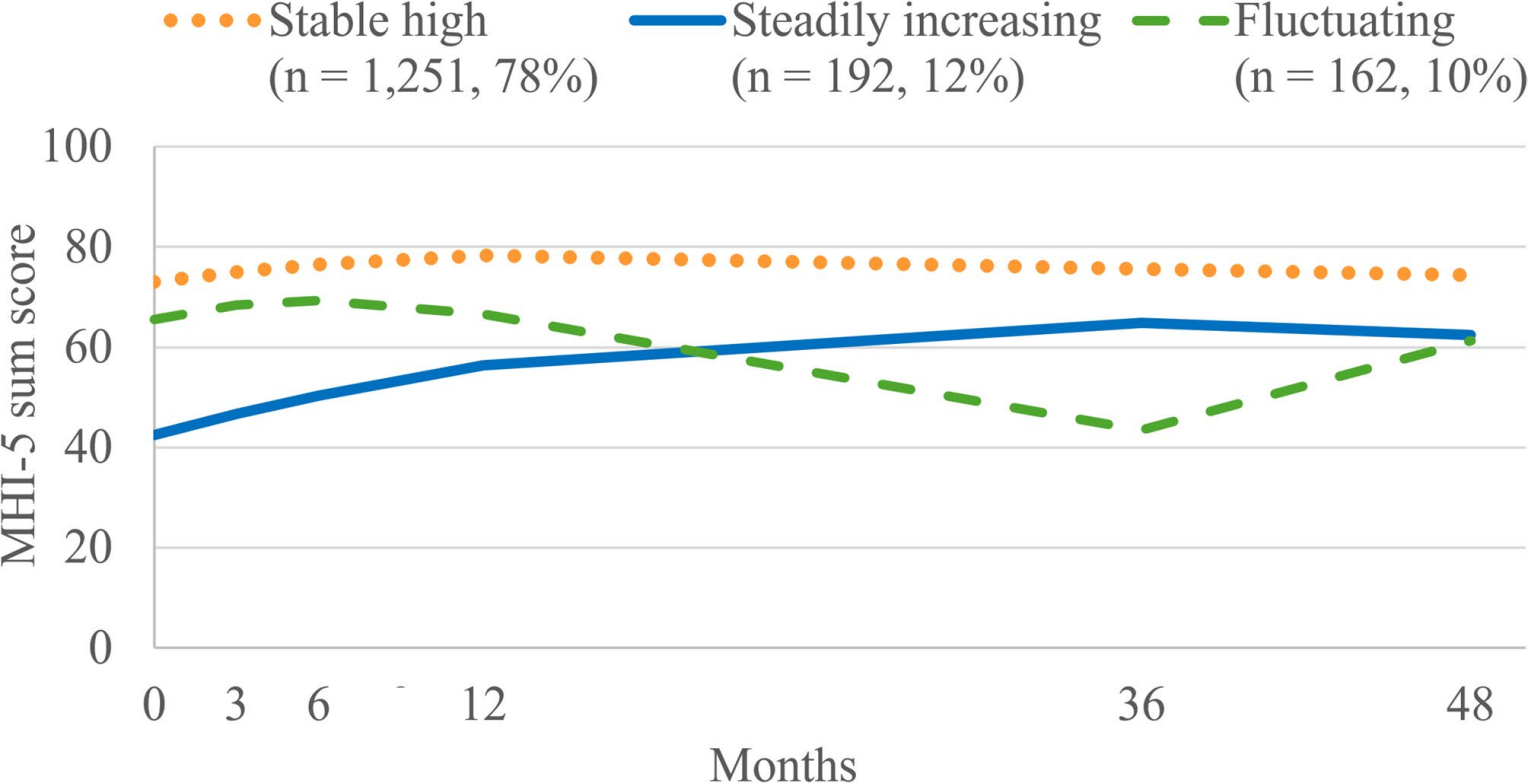
Three latent trajectory classes of mental well-being. *Note*. MHI-5 = Mental Health Inventory

Three distinct latent trajectory classes of mental well-being were identified. The graphical representation of these three latent trajectory classes is illustrated in Fig. 2. For comparison, the model with four latent trajectory classes is described briefly in Supplement 1. The first class (*n* = 1,251, 78%), labeled as “stable high”, was characterized by the highest mental well-being throughout the study with only minor fluctuations over time. The second class (*n* = 192, 12%) is characterized by an average MHI-5 sum score of 42.5 at baseline, which is the lowest MHI-5 sum score of the three classes at baseline. Then, the MHI-5 sum score increased over time reaching its plateau at 36 and 48 months. This latent trajectory class was therefore labeled “steadily increasing”. The third class (*n* = 162, 10%) was characterized by a “fluctuating” trajectory. At baseline, the model-implied MHI-5 score for this class was 65.5, followed by a slight increase peaking at 6 months. This development was followed by a steady decline to the bottom level of mental well-being, which was reached at 36 months and then increased back to almost the baseline level at 48 months.

**Table 1:** Sample characteristics

Study variables	*n* (%)	M (SD)	Median
Sociodemographic variables			
Sex			
Male Female	712 (44%) 893 (56%)		
Age in years		31.04 (10.83)	28.00
School education			
9 years or less 10–11 years 12 years or more	96 (6%) 458 (29%) 1051 (65%)		
Being in a relationship			
Yes No	1,052 (66%) 553 (34%)		
Health behaviors			
Current smokers	509 (32%)		
AUDIT-C		3.51 (1.79)	3.00
Portions of fruit and vegetables per day		2.17 (1.44)	2.00
Minutes of MVPA per day		73.55 (81.23)	50.00
Mental well-being			
MHI-5 sum score baseline (*n* = 1,162) MHI-5 sum score 3 months (*n* = 1,407) MHI-5 sum score 6 months (*n* = 1,334) MHI-5 sum score 12 months (*n* = 1,314 MHI-5 sum score 36 months (*n* = 1,074) MHI-5 sum score 48 months (*n* = 975)		66.81 (15.90) 72.42 (14.28) 73.32 (14.56) 73.87 (14.57) 71.60 (14.40) 72.12 (14.56)	70.00 75.00 75.00 75.00 75.00 75.00

*Note. N* = 1,605, unless otherwise specified. *M* = mean, *SD* = standard deviation. AUDIT-C = Alcohol Use Disorders Identification Test - Consumption. MVPA = moderate-to-vigorous physical activity. MHI-5 = Mental Health Inventory

**Table 2:** Comparison of GMMs with different numbers of latent trajectory classes

	2 classes	3 classes	4 classes^1^	5 classes^1^
Entropy	0.874	0.782	0.802	0.821
LCPP	0.865	0.833	0.757	0.739
BIC	56592.488	56456.164	56377.519	56316.237
AIC	56495.632	56332.404	56226.855	56138.668
VLMR *p*-value	< 0.001	0.009	0.245	0.674
Class sizes	219 (14%) 1,386 (86%)	192 (12%) 1,251 (78%) 162 (10%)	63 (4%) 159 (10%) 160 (10%) 1,223 (76%)	73 (5%) 150 (9%) 1,207 (75%) 144 (9%) 31 (2%)

*Note*. LCPP = lowest average latent class posterior probability. BIC = Bayesian Information Criterion. AIC = Akaike Information Criterion. VLMR = Vuong-Lo-Mendell-Rubin Likelihood Ratio Test. ^1^ For the GMMs with 4 and 5 latent trajectory classes, the best loglikelihood values could not be replicated

### Covariates of latent trajectory class membership

The odds of being classified into the “steadily increasing” (*OR* = 0.46, *95% CI* = [0.29; 0.73]) or “fluctuating” (*OR* = 0.42, *95% CI* = [0.24; 0.72]), compared to the “stable high” class were higher for men than for women (Table 3). Lower age increased the odds of being classified into the “steadily increasing” (*OR* = 0.97, *95% CI* = [0.95; 0.99]) or “fluctuating” (*OR* = 0.97, *95% CI* = [0.95; 0.99]), compared to the “stable high” class. The odds of being assigned to the “steadily increasing” compared to the “stable high” (*OR* = 1.94, *95% CI* = [1.20; 3.13]) or “fluctuating” (*OR* = 1.98, *95% CI* = [1.01; 3.90]) class were higher for participants with more than 12 years of school education than for participants with less than 12 years of school education. The odds of being assigned to the “steadily increasing” compared to the “stable high” class were higher for participants being in a relationship (*OR* = 2.32, *95% CI* = [1.52; 3.54]) than for participants not being in a relationship. Health behaviors (smoking, alcohol consumption, fruit and vegetable intake, moderate-to-vigorous physical activity) did not significantly predict any latent trajectory class membership.

**Table 3:** Results of the multinomial regression analysis predicting latent trajectory class membership

	Steadily increasing vs. Stable high		Steadily increasing vs. Fluctuating		Fluctuating vs. Stable high	
	*OR*	*95% CI*	*OR*	*95% CI*	*OR*	*95% CI*
Sociodemographic variables						
Sex (Ref.: Male)						
Female	**0.46**	**(0.29; 0.73)**	1.10	(0.56; 2.17)	**0.42**	**(0.24; 0.72)**
Age	**0.97**	**(0.95; 0.99)**	0.99	(0.97; 1.02)	**0.97**	**(0.95; 0.99)**
School education (Ref.: < 12 years)						
12 years or more	**1.94**	**(1.20; 3.13)**	**1.98**	**(1.01; 3.90)**	0.98	(0.55; 1.74)
Being in a relationship (Ref.: No)						
Yes	**2.32**	**(1.52; 3.54)**	1.41	(0.75; 2.62)	1.65	(0.96; 2.85)
Health behaviors						
Fruit and vegetable consumption Portions per day	1.11	(0.92; 1.35)	1.08	(0.85; 1.37)	1.03	(0.88; 1.21)
Alcohol consumption						
AUDIT-C sum score	0.94	(0.82; 1.07)	0.86	(0.71; 1.02)	1.10	(0.95; 1.27)
Physical activity						
Daily MVPA per minutes	1.00	(1.00; 1.00)	1.00	(0.99; 1.00)	1.00	(0.99; 1.00)
Current smoking (Ref.: No)						
Yes	0.65	(0.42; 1.01)	0.94	(0.48; 1.85)	0.69	(0.37; 1.28)

*Note*. All regressions were adjusted for study group assignment. Ref. = reference group. AUDIT C = Alcohol Use Disorders Identification Test - Consumption. MVPA = moderate-to-vigorous physical activity. Numbers in bold are statistically significant at *p* < 0.05

## Discussion

In a German general population sample, three latent classes with distinct trajectory patterns of mental well-being were found over a four-year observation period: a “stable high”, a “steadily increasing” and a “fluctuating” trajectory class. Multinomial logistic regression analysis revealed that sex, age, school education and being in a relationship predicted latent trajectory class membership, whereas the health behaviors (smoking, alcohol consumption, fruit and vegetable intake, moderate-to-vigorous physical activity) did not.

The “stable high” trajectory, the largest group showed consistently high MHI-5 values with sum scores ranging between 73 and 78 over time. Thus, the majority of participants showed a high level of mental well-being over a period of four years. The “steadily increasing” trajectory was characterized by a baseline MHI-5 sum score of 43 indicating low mental well-being at the beginning of the study. Their mental well-being increased continuously to an MHI-5 sum score of 65 three years later, after which it slightly decreased to 62 at 48 months. The “fluctuating” trajectory was the only pattern that had pronounced increasing, as well as decreasing trends in the MHI-5 sum score over time. Both the “steadily increasing” and “fluctuating” trajectory included time points, where the model-estimated MHI-5 sum scores fell below the cut-off values for mood and anxiety disorders [45], suggesting that individuals assigned to these trajectory patterns experienced periods of significant and maybe clinically relevant psychological distress. The “steadily increasing” and “fluctuating” courses differed substantially from the average observed MHI-5 trajectory of the overall sample. This underscores the value of mixture modeling, since relying on the mean observed values over time may obscure highly relevant, unique trajectory patterns.

The three latent trajectory classes may reflect distinct dynamics of mental well-being, which may be influenced by specific characteristics of the sample and psychosocial or contextual factors. For example, the “stable high” trajectory may reflect individual resilience and resources, supportive or favorable environmental conditions, and high mental well-being that is less susceptible to external stressors. The “steadily increasing” trajectory, although showing an improvement over time, had substantially lower mental well-being than the “stable high” trajectory throughout the study. This trajectory might reflect impaired mental health at the beginning of the study, followed by an active or passive adaptation process, leading to a continuous reduction of psychological distress. Whereas this latent trajectory class only included a mere 12% of the general population sample under study, trends of increasing mental well-being at the population level have been shown previously [55]. The “fluctuating” trajectory could be indicative of unstable life circumstances or recurring challenges. It is plausible that participants assigned to the “fluctuating” trajectory may have been particularly vulnerable to the disruptions caused by the COVID-19 pandemic, which had its onset in the middle between the 12- and 36-month assessment of our study. The “fluctuating” trajectory might be indicative of the burden imposed by social (e.g. isolation) or economic (e.g. unemployment, income changes) strains. Due to the rapidly evolving intensity of containment measures, especially during the initial phase of the pandemic, in conjunction with the ongoing data assessment that spanned approximately three months for each wave, and the substantial time interval between the 12- and 36-month assessment, conclusions regarding the pandemic’s impact on the study findings remain highly speculative. Nevertheless, it has been shown that the COVID-19 pandemic decreased mental health in Germany [56]. A longitudinal study of mental well-being conducted in Denmark just before and during the COVID-19 pandemic demonstrated a slight decrease in mental well-being from 2019 to 2020. After that, a modest uptick was observed from 2020 to 2021, though it remained below the 2019 baseline. The magnitude of this fluctuation was particularly pronounced among women, individuals under the age of 75, and those with the highest level of education [57]. The Danish study indicated a decline in mental well-being with a slight recovery in 2021, maybe resembling the “fluctuating” trajectory identified in the present sample. The “stable high”, “steadily increasing”, and “fluctuating” trajectories of mental well-being found in this study emphasize that mental well-being can follow different trajectories over time, thereby highlighting its dynamic nature.

The analysis showed that women were about twice more likely than men to be classified into the “stable high” trajectory compared to the “steadily increasing” or “fluctuating” trajectory. Older participants were also more likely to show a trajectory of “stable high” rather than a “steadily increasing” or “fluctuating” trajectory of mental well-being. The likelihood of being assigned to the “steadily increasing” or “fluctuating” trajectory compared to the “stable high” trajectory decreased by three per cent with each year of age. Participants with 12 or more years of school education were almost twice as likely classified into the “steadily increasing” trajectory compared to the “fluctuating” or the “stable high” trajectory, respectively. Participants currently in a relationship were more than two times more likely to report a “steadily increasing” trajectory of mental well-being compared to those not in a relationship. Based on transformed benchmarks for effect sizes according to Bornstein et al. [58], these effects can be considered small to medium, reflecting prior studies that demonstrated the role of sociodemographic factors in differences in mental well-being [7, 30, 32–34, 59]. Potential explanations include mechanisms such as differences in resource availability, resilience, social roles, life experiences, individual stressors, and coping strategies [60, 61].

The identification of distinct trajectories of mental well-being and their sociodemographic predictors might have implications for targeted prevention and public health measures. Although previous research showed women to be at higher risk for mental health problems [15, 35], women were more likely to maintain “stable high” mental well-being. This suggests that various sex- and gender-specific risk and protective factors exist for mental well-being, that could be identified and taken into account in intervention design. The finding that older age predicted more stable mental well-being trajectories supports the value of life-course approaches to mental health promotion. The relationship between higher school education and the “steadily increasing” trajectory suggest that targeted support during educational transitions may be beneficial, especially to university students who may be exposed to high demands at the beginning of and during their studies. Prevention based in educational settings may include mental health monitoring and support programs, particularly during periods of high academic stress. On the other hand, older age and higher education are generally more likely to be associated with better access to resources, a higher level of resilience, enhanced professional and social opportunities, as well as improved access to health information, which may be beneficial for mental well-being [38, 61]. Whereas social support and connectedness may bear a positive effect on mental well-being trajectories, the finding that being in a relationship lowered the probability for stable-high mental well-being is unexpected. In the literature, romantic partnerships have been shown to exert a dual influence on mental health, functioning as both a protective and risk factor, depending on individual needs and the quality of the relationship [32]. Future research should explore the role of subjective quality of social relationships and the mechanisms behind the effect of school education on mental well-being trajectories. This could provide deeper insights into how interpersonal dynamics and socio-economic status contribute to mental well-being.

Past research also suggested that health behaviors can have both a positive and negative impact on mental well-being [15–18]. In the present study, health behaviors did not predict latent mental well-being trajectory class membership. It is possible that the effect of health behaviors is small, and our logistic regression lacked sufficient statistical power. The finding that health behaviors were not significantly associated with latent class membership may be attributed to further methodological circumstances. Health behaviors were assessed with a small number of items, potentially limiting the reliability of measurement. The applied assessment of health behaviors may not have been capable of differentiating the full variety within each behavior. For example, the self-report data on physical activity did not allow for differentiation of varying levels of intensity, thus preventing a more precise operationalization of participants’ physical activities, for example through metabolic equivalents. Similarly, the self-reported consumption of fruits and vegetables does not fully reflect dietary habits, potentially leading to a homogenization of the data and compromised validity. It is conceivable that participants who have previously smoked may have ceased to do so as a consequence of health concerns pertaining to their mental well-being, a factor which could potentially influence the observed associations. Additionally, the instruments utilized to assess health behaviors were all based on self-reports.

The application of GMM allowed us to account for unobserved sample heterogeneity and to identify different longitudinal patterns of similar trajectories of mental well-being over time. Since GMM is an exploratory statistical procedure, the findings should be considered as hypothesis-generating for future studies. The latent trajectory classes are described by model-implied means, while permitting within-class variation through random growth factor variances. That means that there remains considerable variation in individual mental well-being trajectories within each latent class. Missing data due to nonparticipation in follow-up assessments was accounted for by state-of-the-art full-information maximum likelihood estimation, which assumes that data are missing at random. Study attrition may be dependent on how participants’ health status develops over time [62]. Whereas loss to follow-up in the PRINT study was associated with age, school education, smoking, and alcohol use at baseline [41], it is impossible to determine if data are missing at random or not at random [63]. Therefore, bias through non-ignorable missingness cannot be ruled out.

### Strengths and limitations

The present study followed a general population sample over a period of four years and contributed to the existing literature regarding temporal patterns of mental well-being. We added to the research about the MHI-5 as a continuous measure of mental well-being. As screening results are usually from one point of time [60, 64], there was a lack of knowledge regarding differential trajectories. The following limitations have to be acknowledged: All participants were recruited in Greifswald, a town of 60,000 residents in north-east Germany that is characterized by a large proportion of university students and employees. Participants had to have consumed alcohol at least once in the twelve months prior to the baseline assessment and have sufficient proficiency in the German language. Thus, the generalizability of the findings to the general population may be limited, in particular to alcohol consumers, other and more rural areas of Germany and to individuals who speak little or no German. The intervention tested in the PRINT trial was designed as a low-threshold brief intervention with the primary objective of enhancing motivation to reduce participants’ alcohol consumption. We found no evidence for intervention efficacy [43]. Consequently, it appears reasonable to assume that the intervention did not exert a systematic influence on the development of mental well-being among the participants randomized to the intervention group. However, this cannot be ruled out completely. The use of self-report in this context carried the potential for the elicitation of socially desirable responses or the occurrence of recall bias. The MHI-5 measures mental well-being during the last month. Short-term changes and fluctuations of mental well-being might have been overlooked, since there were at least three months between each assessment. To uncover these short-term changes, a more fine-grained study design would have been necessary. As this study is a secondary data analysis, mental well-being was only assessed six times during the four-year study period. What is more, important covariates for mental well-being trajectories such as (family) history of or current mental disorders, physical health (e.g. chronic diseases), socio-economic factors (e.g. income, unemployment), or the participants’ personality [65] could not be incorporated into the analysis, as they were not assessed in the trial. While the identification of latent trajectory classes of mental well-being would have remained unchanged with the inclusion of further covariates due to our statistical three-step approach, our findings are limited as it is not possible to draw conclusions on how the important missing determinants of mental well-being might have influenced the assignment of participants to the three latent trajectory classes.

## Conclusion

Three different temporal patterns of participants’ mental well-being were identified in a German general population sample, with the majority showing a stable level of high mental well-being over a four-year period. The study draws attention to the notion that mental well-being is dynamic and influenced by a variety of factors. Further research is needed to understand fluctuating patterns and the causal factors influencing mental well-being. As only associations have been identified so far, future studies should attempt to disentangle causal relationships between health-related behaviors and mental well-being. Moreover, further studies could encompass extended time periods in order to enhance comprehension of the longitudinal changes in mental well-being, so that future findings can inform individual and societal interventions to promote mental well-being.

## Electronic supplementary material

Below is the link to the electronic supplementary material.

Supplementary Material 1

## Data Availability

The data sets used and analyzed during the current study are available from the corresponding author on reasonable request.

## Abbreviations

AUDIT-C: Alcohol use disorders identification test-consumption
CI: Confidence interval
DRKS: German register of clinical trials
GMM: Growth mixture model
M: Mean
MHI-5: Mental health inventory-5
MVPA: Moderate-to-vigorous physical activity
OR: Odds ratio
SD: Standard deviation
