# Supplementary material for: Longitudinal patterns of mental well-being over four years in a german general population sample: a growth mixture modeling approach
Source: BMC Public Health. 2025 Jun 26;25:2175. doi: 10.1186/s12889-025-23539-w (PMC12199513; doi:10.1186/s12889-025-23539-w)
Supplement: Supplementary file 1 — Supplementary Material 1 [file 12889_2025_23539_MOESM1_ESM.docx]

**Supplement 1**

In this Supplement, the Growth Mixture Model with four latent trajectory classes is described. It has to be noted that the best loglikelihood value for this model could not be replicated, even with a high number of random starting values. This means that the results may not be trustworthy due to local maxima.

The model with four latent trajectory classes (Figure S1) yielded three classes that were very similar to the main results: “stable high”, “steadily increasing”, and “fluctuating”. The additional fourth latent trajectory class (*n* = 63, 4%) was characterized by consistently lower MHI-5 sum scores compared to the other three latent trajectory classes. Therefore, this class was labeled “highly burdened”.

**Figure S1:** Four latent trajectory classes of mental well-being


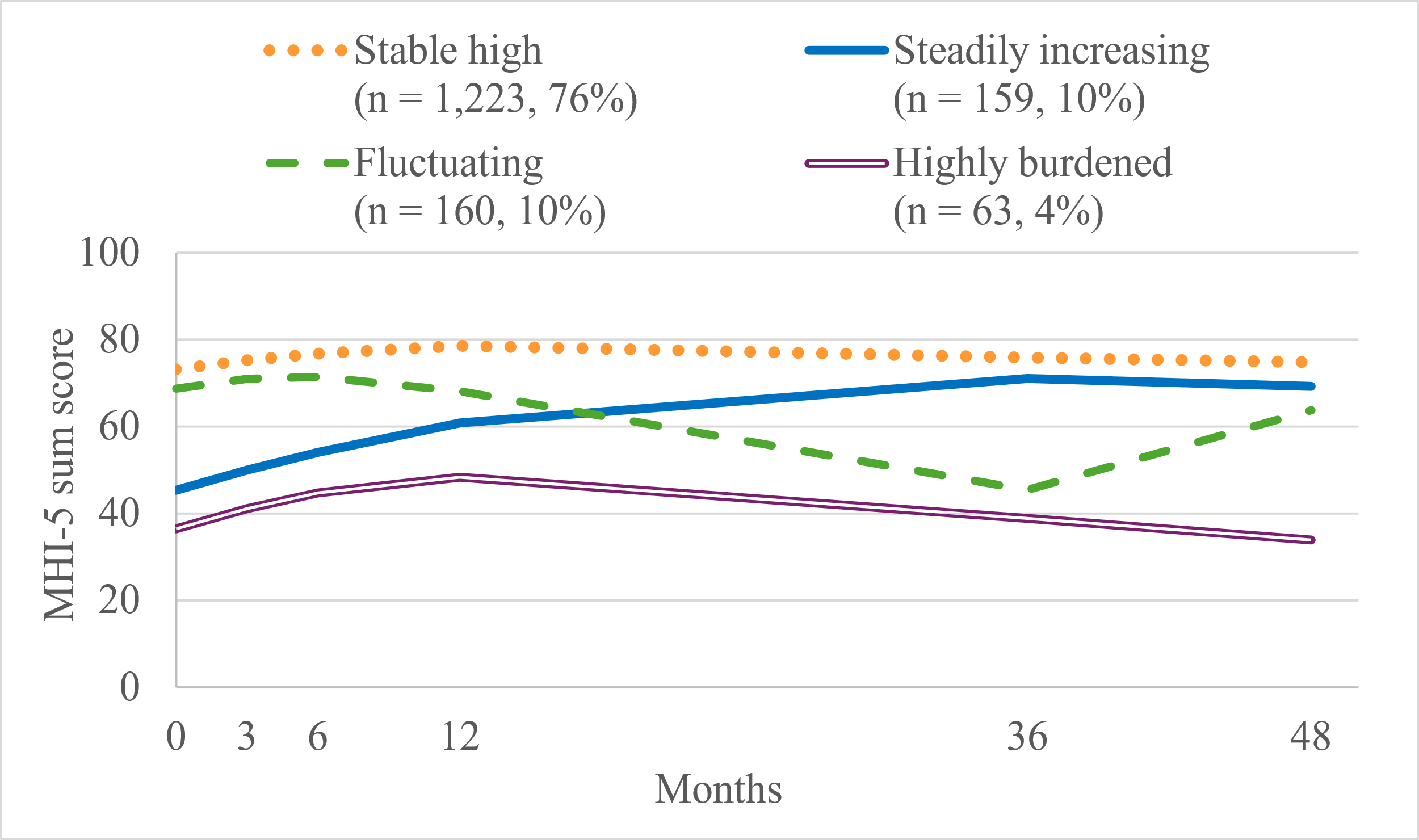


*Note.* MHI-5 = Mental Health Inventory.
